# Carrier Dynamics of Efficient Triplet Harvesting in AgBiS_2_/Pentacene Singlet Fission Solar Cells

**DOI:** 10.1002/advs.202300177

**Published:** 2023-03-20

**Authors:** Pai Geng, Dezhang Chen, Sunil B. Shivarudraiah, Xihan Chen, Liang Guo, Jonathan E. Halpert

**Affiliations:** ^1^ Department of Chemistry Hong Kong University of Science and Technology Clear Water Bay, Kowloon Hong Kong 999077 Hong Kong SAR; ^2^ Department of Mechanical and Energy Engineering Southern University of Science and Technology Shenzhen 518055 P. R. China; ^3^ Key Laboratory of Energy Conversion and Storage Technologies (Southern University of Science and Technology) Ministry of Education Shenzhen 518055 P. R. China; ^4^ SUSTech Energy Institute for Carbon Neutrality Southern University of Science and Technology Shenzhen 518055 P. R. China; ^5^ Energy Institute The Hong Kong University of Science and Technology Clear Water Bay Kowloon Hong Kong 999077 China; ^6^ State Key Laboratory on Advanced Displays and Optoelectronics Technologies Department of Electronics and Computer Engineering The Hong Kong University of Science and Technology Kowloon Hong Kong 999077 China

**Keywords:** AgBiS_2_ nanocrystal, charge transfer, chromophores, singlet fission, solar cells

## Abstract

Singlet fission is a process by which an organic semiconductor is able to generate two triplet excitons from a single photon. If charges from the triplets can be successfully harvested without heavy losses in energy, then this process can enable a single‐junction solar cell to surpass the Shockley–Queisser limit. While singlet fission processes are commonly observed in several materials, harvesting the resulting triplets is difficult and has been demonstrated with only a few transport materials. Here, transient absorption spectroscopy is used to investigate singlet fission and carrier transfer processes at the AgBiS_2_/pentacene (AgBiS_2_/Pc) heterojunction. The successful transfer of triplets from pentacene to AgBiS_2_ and the transfer of holes from AgBiS_2_ to pentacene is observed. Further singlet fission in pentacene by modifying the crystallinity of the pentacene layer and have fabricated the first singlet fission AgBiS_2_/Pc solar cell is enhanced. Singlet fission devices exhibit higher external quantum efficiency compared with the control devices, and thus demonstrating the significant contribution of charges from the singlet fission process.

## Graphical abstract

In this study, a singlet fission solar cell is designed with pentacene and AgBiS_2_ nanocrystals. Both high efficient singlet fission and stable separation of carriers are explored by transient absorption spectroscopy. An annealing process is adopted to further promote singlet fission. Finally the singlet fission devices exhibit high external quantum efficiency and near‐unity internal quantum efficiency (IQE), and the isolated IQE up to 160% from pentacene layer is achieved.

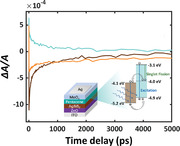

## 1 Introduction

Singlet fission (SF) is a spin‐allowed process in which internal conversion occurs from a high‐energy excited singlet state to two low‐energy triplet states.^[^ ^1^ ^]^ By reducing thermalization losses,^[^ ^2^ ^]^ the SF process has been predicted to boost the solar cell conversion efficiency, theoretically enabling single junction cells to overcome the ey–Queisser efficiency limit.^[^ ^3^, ^4^ ^]^ Thus photovoltaic devices using SF and the charge generation and transport mechanisms in these devices have recently attracted significant attention.^[^ ^5^, ^6^ ^]^ Tayebjee et al. calculated the theoretical limit of the efficiency of SF devices to be 45.9% at 300 K and under AM1.5G illumination, almost 50% higher than the maximum efficiency of a normal single junction solar cell (33.7%).^[^ ^7^ ^]^ Among several molecules that exhibit SF, the organic semiconductor pentacene (Pc) is one of the most popular candidates for SF solar cells, due to the rapid (< 100 fs), high‐yield triplet generation observed in Pc under solar‐spectrum illumination.^[^ ^8^, ^9^ ^]^ However, simply using SF chromophores as the solar cell active layer is insufficient to achieve solar cell improvement since the doubled photocurrent would also bring a halved voltage of the cell and thus no benefit to the peak power.^[^ ^3^ ^]^ Instead a suitable triplet acceptor must be chosen that sets the active layer bandgap, harvests high energy photons as SF triplets while also directly converting lower energy (but still above *E*_g_) photons to normal photocurrent.

In recent years, several attempts have been made to use SF in optoelectronic applications. Ehrler et al. first reported an SF solar cell in 2012 with Pc as the SF chromophore and PbSe nanocrystals (NCs) as the triplet acceptor.^[^ ^5^ ^]^ Macqueen et al. fabricated a tetracene/Si solar cell in 2018, but the endothermic SF process slowed triplet generation, and the low quantity of triplets reduced the benefit of using the SF mechanism.^[^ ^10^ ^]^ Einzinger et al. utilized electric‐field‐effect passivation to achieve an efficient energy transfer of triplets generated in tetracene, further revealed the potential of SF to increase the efficiency of Si solar cells.^[^ ^11^ ^]^ Lu et al. reported SF sensitized by CsPbBr_3_ NCs, where triplets were generated efficiently but energy transfer between the CsPbBr_3_ NCs and TIPS‐pentacene exhibited low efficiency, which was attributed to poor wave function overlap between the two materials.^[^ ^12^ ^]^ Guo et al. reported high‐speed triplet electron transfer (<1.5 ps) in a TIPS‐pentacene/MAPbI_3_ heterojunction film, but with much slower hole transport (≈13.8 ns).^[^ ^13^ ^]^

Several factors still hinder device performance including: the relative absorption and luminescence spectra of the materials, the energy difference between *E*_S1_ and 2*E*_T1_, the overlap between the wave functions of the triplet donor and acceptor materials and the stability of chromophore and acceptor material, in addition to other normal device considerations.^[^ ^3^ ^]^ Although Pc exhibits efficient SF under solar illumination, the low *E*_T1_ (0.86 eV) makes it incompatible with common semiconductors, most notably silicon and lead halide perovskites. One unexplored option, AgBiS_2_ nanocrystals are actually promising candidates for this role, due to their matching band energies.^[^ ^14^, ^15^, ^16^, ^17^ ^]^ Besides maintaining good stability, solar cells produced this non‐toxic material also exhibit strong light‐harvesting ability and relatively high power conversion efficiency (PCE).^[^ ^14^, ^18^ ^]^

In this work, we investigate carrier transfer between Pc and AgBiS_2_ nanocrystals and fabricate devices to demonstrate SF and triplet transfer in these materials. Using transient absorption spectroscopy (TAS) measurements we observe that triplets generated in Pc are efficiently transferred to the AgBiS_2_ NC layer. Annealing the Pc layer further promotes the SF process and provides more photocurrent due to an increase in the diffusion length of the triplets. AgBiS_2_/Pc SF solar cells were fabricated with internal quantum efficiency (IQE) of nearly 100%, and up to 160 ± 25% in the Pc layer alone, as determined by transfer matrix optical modelling.^[^ ^19^ ^]^ These results demonstrate the possibility of boosting device performance by employing SF chromophores in AgBiS_2_/Pc solar cells, and provides new ideas for fabricating high‐efficiency nanocrystal solar cells.

## 2 Results and Discussion

During photoexcitation, photoabsorption by Pc causes the generation of singlet excitons

(1) $$S_{1}+S_{0}\underset{k_{2}}{\overset{k_{-2}}{⟷}}{}^{1}\left(TT\right)\underset{k_{1}}{\overset{k_{-1}}{⟷}}T_{1}+T_{1}$$

**Figure 1:**
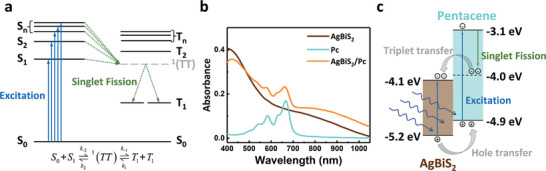
a) A schematic illustration of the singlet fission process in pentacene. b) The UV–vis spectra of AgBiS_2_, pentacene, and AgBiS_2_/Pc heterojunction, and c) a schematic illustration of the charge transfer process between AgBiS_2_ and pentacene.

Equation (1) describes the simplified SF process (**Figure** **1**a) and its reverse process, triplet–triplet annihilation (TTA), where *S*_0_ is the ground state of the chromophore, *S*_1_ and *T*_1_ are the lowest‐energy excited singlet and triplet states, respectively,^1^(*TT*) represents a coupled triplet pair, and *k* represents the rate constant for each step.^[^ ^20^ ^]^ AgBiS_2_ NCs with an average diameter of ≈5 nm were synthesized by adapting a previously reported hot‐injection method (Figure S1, Supporting Information).^[^ ^14^ ^]^ AgBiS_2_ NCs thin films were formed by spin‐coating the NC solution onto the substrate. Pc films were formed by thermal evaporation (see the Supporting Information for details). Figure 1b shows the UV–Vis–NIR absorption spectra of those samples. As a narrow‐band semiconductor with an indirect band gap at ≈1.1 eV, AgBiS_2_ NCs have a wide absorption feature covering the visible to NIR range (400–1100 nm). In contrast, Pc only shows strong absorption at wavelengths below 700 nm, corresponding to its wider effective band gap at ≈1.8 eV. The composite sample exhibits the characteristics of the two materials, with a wide absorption range and small absorption peaks visible at 584 and 666 nm. Since the photoexcitation from the singlet ground state to a triplet state is spin‐forbidden, no corresponding absorption peak can be collected in the UV–Vis–NIR absorption spectra.

According to UV‐photoelectron spectroscopy (UPS), the highest occupied molecular orbital (HOMO) energies of AgBiS_2_ NCs and Pc are −5.2 and −4.9 eV, respectively (Figure S2, Supporting Information). Combined with their reported band gaps, we propose a carrier transfer model for the AgBiS_2_/Pc heterojunction (Figure 1c). Triplets generated by the SF process in Pc accumulate at about −4.0 eV and are then transferred into the conduction band of AgBiS_2_ at −4.1 eV. Holes generated in AgBiS_2_ are transferred into the Pc, and the spin‐forbidden triplet recombination promotes retention of the separated carriers. The increase in excited electrons in the NC film, up to 200% of the photons absorbed by the Pc layer, permits the total IQE to rise above 100%.

**Figure 2:**
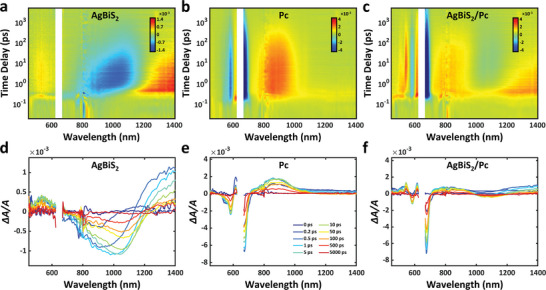
TA maps of a) AgBiS_2_, b) Pc, and c) AgBiS_2_/Pc. The evolution of TA spectra with different time delay for d) AgBiS_2_, e) Pc, and f) AgBiS_2_/Pc, respectively. Signals with probe wavelengths in the range of 625–660 nm are discarded due to spectral overlap with the pump laser whose wavelength is 650 nm. a–c) have independent scales, d–f) share a set of timelines shown in (e).

TAS was performed to observe charge dynamics in Pc and NC bi‐layer films. **Figure** **2** shows the evolution of the transient absorption (TA) signal for AgBiS_2_, Pc, and AgBiS_2_/Pc samples. A 650 nm pump laser, which can excite both the AgBiS_2_ and Pc layers, was employed for all samples with 1 kHz repetition rate to avoid heat accumulation. A broadband ground state bleaching (GSB) signal centered around 1050 nm was observed in the AgBiS_2_ sample (Figure 2d), corresponding to the narrow indirect band gap observed in the UV–vis–NIR absorption spectrum (Figure 1b). Meanwhile an apparent photo‐induced absorption (PIA) signal can be observed via a longer probe wavelength (>1200 nm). The Pc sample presents two sharp GSB features at 584 and 666 nm, corresponding to– the S_0_ → S_1_ and S_0_ → S_2_ electronic transition, respectively (Figure 2e).^[^ ^21^ ^]^ Two PIA signals at around 620 and 700–1100 nm can be explained by the existence of singlets and triplets in the Pc, and the singlet PIA features shows some overlap in wavelength with the GSB signal. Singlets with high energy and short lifetimes result in a quick decay of the PIA signal at 600 nm,^[^ ^22^ ^]^ while the low‐energy triplets with long lifetime dominate the slow decay of the PIA signal at 700–1100 nm. Their larger overall absorbance of the probe results in a stronger PIA signal than the singlet PIA.

**Figure 3:**
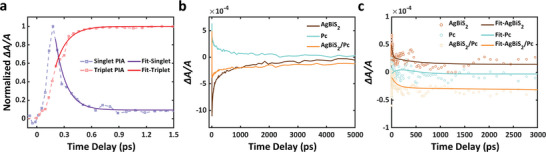
a) PIA signals of singlets (purple) and triplets (red) in Pc. Probed with 607 and 872 nm, respectively. The solid curves are the fitting results with single exponential functions. b) TA spectra for all samples with 650 nm pump laser and 1063 nm probe laser. c) TA spectra for all samples with 750 nm pump laser and 690 nm probe laser.

For the AgBiS_2_/Pc composite sample (Figure 2f), both the GSB and the PIA signals of Pc are clearly exhibited. In the NIR region (800–1200 nm), the AgBiS_2_ GSB signal is diminished, this can be attributed to the carrier transfer process that the holes generated in AgBiS_2_ transfer into the valence band of Pc. The increased electron density in the valence band of AgBiS_2_ brings an increased absorption of the probe laser, leading to a suppressed GSB signal. However, at long time delay, the triplets generated in Pc will transfer to the conduction band of AgBiS_2_, leading to an enhanced GSB signal around the band gap. Our results match previous observations of the SF process in Pc and confirm the carrier transfer to AgBiS_2_ as shown in **Figure** **3**.

When *E*_S1_ > 2*E*_T1_, as in Pc, SF is both energetically and entropically favorable, and this results in a rapid triplet generation process ultrafast timescale, outcompeting the radiative decay of the singlet.^[^ ^3^, ^23^ ^]^ Figure 3a presents the PIA features of Pc due to singlets and triplets, respectively. Because the singlet with a short lifetime exhibits the PIA feature, and this shows obvious spectral overlap with the GSB of Pc, the singlet PIA signal is collected at the node of the spectra at 1 ps to remove the contribution of the GSB signal. Note that the singlet PIA and the triplet PIA features demonstrate a pair of decreasing and increasing functions, respectively. We use a pair of single exponential functions to fit the decay and growth processes of the two curves, and get the time constants for these two features (Equations (2) and (3))

(2) Ysinglet=Asinglet·et−t0t−t0τsingletτsinglet+y0,singlet

(3) Ytriplet=1−Atriplet·et−t0t−t0τtripletτtriplet+y0,triplet

where *A* is the amplitude, *τ* is the rise or decay time constant, *t*_0_ is the starting time, *y*_0_ is a constant background signal (see Table S1 for fitting parameters, Supporting Information). The existence of SF in Pc can be confirmed by a pair of similarly valued *τ*_singlet_ and *τ*_triplet_, 118.4 ± 22.1 and 128.4 ± 10.4 fs, respectively, of the fall and rise features of the singlet PIA and the triplet PIA. These two time constants are equivalent within uncertainty while the minor difference between *τ*_singlet_ and *τ*_triplet_ could be attributed to the TTA process and the recombination of few singlets and holes. Both signals stabilized after 1 ps, demonstrating that the SF here is an ultrafast process with a much smaller timescale than conventional carrier transfer, and triplets in Pc are generated within hundreds of femtoseconds and long‐lived thereafter. The kinetics of the singlet PIA peak at 620 nm is also notable (Figure S3, Supporting Information). The ultrafast signal generated in the Pc sample exhibits a sharp decay with a short time delay, and the curve flattens at around 25% of the peak intensity. This strongly suggest that most of the singlets (≈75%) go on to generate triplets in Pc, and the high yield of triplet charges then dominates carrier transfer processes observed in the SF solar cells.

To study the carrier transfer between the two materials, we observed the dynamics of the triplets and the holes generated in Pc and AgBiS_2_, respectively. In Figure 3b, when probed at 1063 nm, triplets in Pc exhibit a long‐lived PIA signal. We note that at early time delay (< 1 ns), the AgBiS_2_ sample exhibits a strong GSB signal, corresponding to its small bandgap, while the AgBiS_2_/Pc composite sample exhibits a much weaker GSB signal. In contrast, at long time delay (> 1 ns), the GSB signal of the AgBiS_2_/Pc sample is stronger than that of the AgBiS_2_ sample. This can be explained by carrier transfer from Pc to AgBiS_2_. The holes generated in the valence band of AgBiS_2_ can transfer into Pc, and the decreased hole density in AgBiS_2_ enhances the absorption of the probe laser, leading to a suppressed GSB signal. Meanwhile, the triplets generated in Pc enter the conduction band of the AgBiS_2_ via Dexter transfer, and the filled orbitals suppress the photoexcitation, resulting in a stronger GSB signal.^[^ ^12^, ^24^ ^]^ Triplets will not undergo spin inversion during Dexter transfer, so the GSB signal of the composite sample remains at a stable intensity after long time delay. Overall, the more intense and longer‐lived GSB signal of the composite sample demonstrates efficient transfer of triplets from Pc to AgBiS_2_. This process can also be confirmed through further observations, as shown in Figure S4 (Supporting Information). There, when probing at 890 nm, the pure Pc sample will display a strong, long‐lived triplet PIA signal. However, in the composite sample, the PIA signal quickly decreases to zero, due to the efficient triplet transfer from Pc to AgBiS_2_. We can also observe enhancement of the AgBiS_2_ GSB signal in the composite sample. For the pure AgBiS_2_ sample, the GSB signal at 1030 nm will disappear within 5 ns, while in the composite sample, the long‐lived triplets from Pc will occupy the conduction band of the AgBiS_2_ NCs, which extending the lifetime and intensity of the GSB. Note that the nanocrystal GSB signal in the composite sample at 1030 nm undergoes a sharp decrease at ≈2 ps, which matches the expected timing of the triplet transfer from Pc to AgBiS_2_ NCs.

The separation of electrons and holes is a key factor in the performance of photovoltaic devices. A 750 nm pump laser was used to observe hole transfer from AgBiS_2_ to Pc (Figure S5, Supporting Information). The extremely low absorbance of Pc at this wavelength demonstrates that the longer‐wavelength pump laser can only excite AgBiS_2_, not Pc, as is consistent with the negligible TA intensity of Pc within the entire detection region. Figure 3c presents the TA spectra with a 690 nm probe, corresponding to the band gap of Pc. Note that the pure AgBiS_2_ sample exhibits a positive PIA feature which is attributed to broadening of the exciton spectrum,^[^ ^25^ ^]^ while the composite sample shows an apparently negative signal at this wavelength, proving the interaction between two layers. We propose that the holes generated in AgBiS_2_ tend to transfer into the valence band of Pc, and the increased hole density in Pc leads to an apparent GSB signal. The long‐lived negative signal indicates that holes can be stored in the Pc, confirming their efficient separation from the electrons. Thus this bi‐layer system can be used in photovoltaic devices for efficiently capturing charges from multiple excitons per photon.

**Figure 4:**
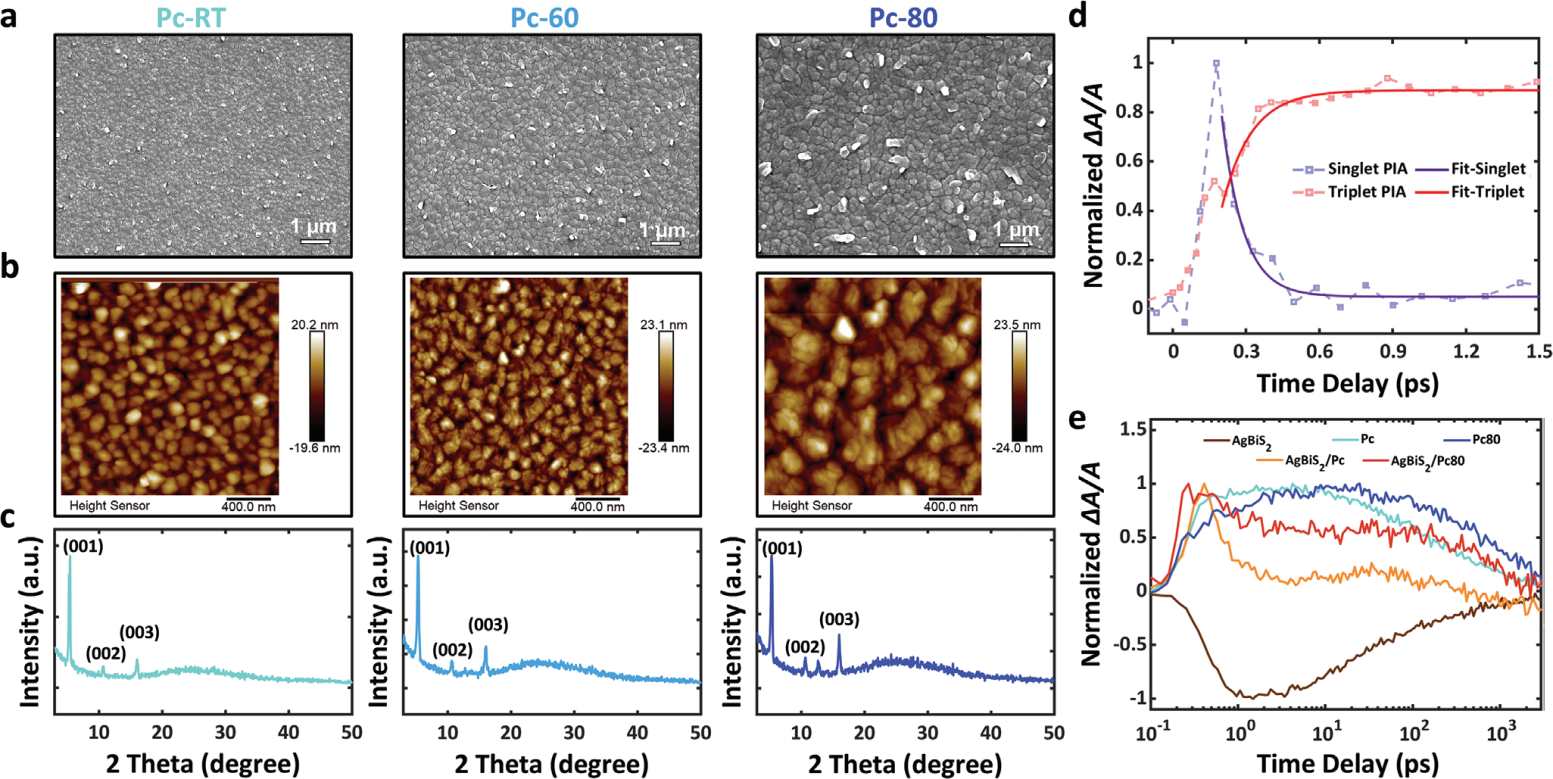
a) SEM image, b) AFM image, and c) XRD pattern of pentacene film annealed under RT, 60 and 80 °C, respectively. d) TAS of the SF process in a Pc film annealed at 80 °C. e) TA spectra for all samples with 650 nm pump and 970 nm probe beams.

Pc films obtained by thermal evaporation are often accompanied by many grain boundaries. It has been reported that the presence of even a small fraction of the amorphous phase (< 10%) will greatly decrease the triplet diffusion length, for example, from 75 to 14 nm in TIPS‐pentacene.^[^ ^26^, ^27^ ^]^ Here we improved the crystallinity of the Pc film by an annealing treatment, reducing the grain boundaries and thereby the trap density. **Figure** **4**a presents the SEM images of three Pc films with different annealing temperatures (room temperature Pc‐RT, 60 °C Pc‐60, 80 °C Pc‐80), and the grain size is observed to increase with annealing temperature. AFM images in Figure 4b reveal the same trend. XRD patterns of different Pc films are shown in Figure 4c. All the samples exhibit characteristic XRD peaks corresponding to (001), (002), and (003), and the Pc‐80 sample shows the best crystallinity, while annealing above 100 °C produces irregular films with small crystallites (Figure S6, Supporting Information).

The carrier dynamics of the annealed sample Pc‐80 and AgBiS_2_/Pc‐80 were also investigated by TAS (Figure S7, Supporting Information), both SF and carrier transfer were observed. Figure 4d presents the PIA features of singlets and triplets in Pc‐80, and the two curves exhibit *τ*_singlet_ and *τ*_triplet_ as 88.1 ± 23.4 and 118.9 ± 36.0 fs, respectively (Table S1, Supporting Information). The smaller *τ* compared with those of Pc‐RT indicates that the better crystallinity of Pc‐80 facilitates the SF process. At longer time delay, the triplets in the annealed samples also exhibit longer lifetimes than those in the samples prepared at room temperature (Figure S8, Supporting Information). Due to the increased density of triplets generated in Pc‐80, carrier transfer between AgBiS_2_ and annealed Pc‐80 is also enhanced. Figure 4e displays the comparison between the carrier dynamics with 650 nm pump and 970 nm probe for all samples. Both the annealed samples (Pc‐80 and AgBiS_2_/Pc‐80) exhibit longer triplet lifetimes.

Although the better crystallinity of Pc should bring a faster carrier transfer, we note that the annealed samples unusually show a slower decay at short time delay. This can be explained by the higher density and the longer diffusion length of the triplets in the annealed sample. The increased density of triplets close to the interface can then transfer to AgBiS_2_, leading to a long‐lived carrier transfer process, and the carriers generated deeper inside Pc‐80 can slowly diffuse to the interface and transfer to AgBiS_2_, further extending the time for PIA signal decay. Overall, the annealing process at 80 °C effectively improves the crystallinity of the Pc film, enhancing SF. The longer diffusion lengths create a long‐lived carrier transfer process between the Pc and the AgBiS_2_ nanocrystals. Thus, triplets and holes are efficiently separated into the AgBiS_2_ and Pc thin films, respectively, indicating the possibility of fabricating a high‐performance SF solar cell.

**Figure 5:**
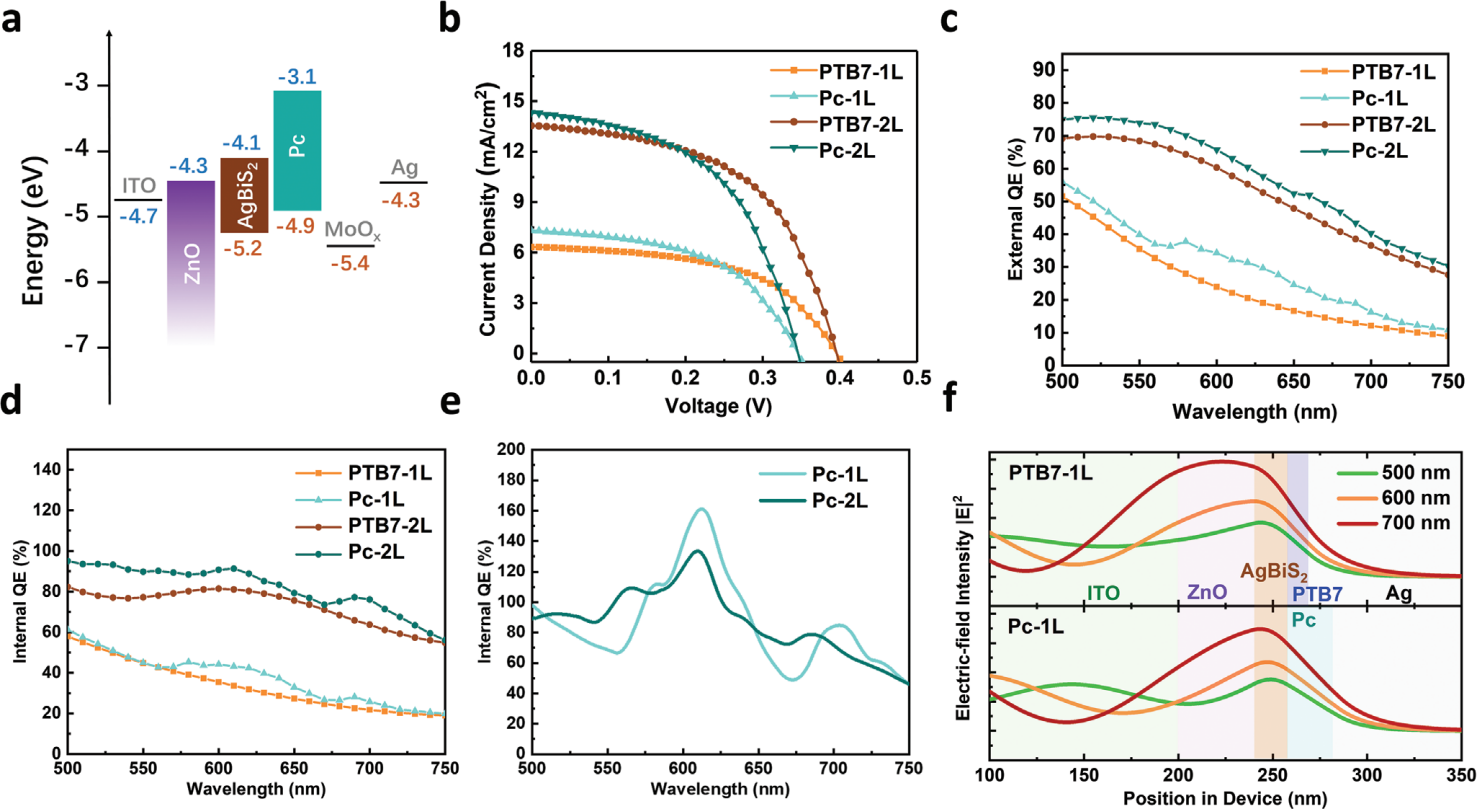
Singlet fission AgBiS_2_ solar cells’ a) band energy diagram. b) *J*–*V* curves of control (PTB7) and singlet fission (Pc) solar cells under 1‐sun illumination, c) EQE curves of solar cells, d) overall IQE curves of solar cells, and e) isolated Pc IQE contribution simulated by transfer matrix optical modeling. f) The simulated interference patterns of 500, 600, 700 nm light in the solar cell devices.

The AgBiS_2_/Pc heterojunction was then incorporated into photovoltaic devices producing what is, to the best of our knowledge, the first SF enhanced AgBiS_2_ solar cells to be reported. The device architecture employed here is an n‐i‐p device type ITO/ZnO NCs/AgBiS_2_/Pc/MoO*_x_*/Ag. The band energy diagram indicates that Pc can be both applied as a hole‐transporting material (HTM) and the SF material (**Figure** **5**a). Since the current contribution from SF depends on active layer thickness,^[^ ^28^ ^]^ the active layer thickness was varied in the devices by depositing one layer (1L) or two layers (2L) of AgBiS_2_. PTB7 is used as the optimized HTM for the control devices based on previously report devices.^[^ ^29^, ^30^ ^]^ The *J*–*V* curve shows that AgBiS_2_/Pc could generates higher short‐circuit current (*J*_SC_) in both the 1L and 2L devices, demonstrating the current contribution from Pc (Figure 5b). The SF devices generate a lower open‐circuit voltage (*V*_OC_) compared with the control devices, which further results in a decreased PCE. The decrease in V_OC_ is attributed to increased nonradiative recombination at the NC/Pc interface for charges originating from the NC layers.^[^ ^31^ ^]^ In Figure 5c, the SF devices have significantly higher EQE in the wavelength range 600–700 nm, corresponding to the Pc absorption peak. SF devices using Pc‐80 slightly improved the *J*_SC_ (Figure S9, Supporting Information). Transfer matrix optical modeling was used to calculate the absorbance fraction in the devices (Figure S10, Supporting Information), which permits calculation of the IQE value for each device.^[^ ^19^ ^]^ For control devices, the IQE was calculated by dividing the EQE by the fraction of photons absorbed by AgBiS_2_ NCs. For SF devices, the overall IQE was calculated by Equation (4)

(4) $${\mathrm{IQE}}_{{\mathrm{AgBiS}}_{2}/\mathrm{PC}}=\frac{{\mathrm{EQE}}_{{\mathrm{AgBiS}}_{2}/\mathrm{PC}}}{{\mathrm{Abs}}_{{\mathrm{AgBiS}}_{2}}+{\mathrm{Abs}}_{\mathrm{PC}}}$$

where Abs_AgBiS2_ and Abs_Pc_ are the absorbed fraction of AgBiS_2_ NCs and Pc in the SF device, respectively.

Figure 5d shows that Pc has a positive overall IQE contribution for both the 1L and 2L SF devices. The IQE contribution from the Pc component is obtained by isolating the AgBiS_2_ EQE contribution in the system (Figure S11, Supporting Information). In the Pc‐1L and Pc‐2L samples, the IQE within the pentacene layer reaches a peak of 160 ± 25% and 130 ± 20%, respectively, at 610 nm (Figure 5e), which demonstrates an efficient SF process is happening in both devices. For photons at 500 and 600 nm, the peak electric field intensity is located in the AgBiS_2_ layer in both the control and SF devices (Figure 5f), indicating an efficient light harvesting in this structure. At 700 nm, the most intense electric field is inside the ZnO layer, which may be the reason for weak exciton generation observed in the EQE spectrum at those wavelengths. Replacement of PTB7 by Pc also enables significantly more efficient light harvesting in the thin layer AgBiS_2_ (≈17 nm). Thicker NC films in this n‐i‐p architecture obscure the SF process and thus further engineering is needed to realize the full potential of SF harvesting in these devices (Supporting Information).

## 3 Conclusion

In conclusion, the carrier transport process in the AgBiS_2_/Pc heterojunction is determined by the band structure, as revealed by transient absorption spectroscopy. SF in pentacene was confirmed to generate triplets on femtosecond scale (≈100 fs), and the transfer of triplet electrons and holes in the Pc/AgBiS_2_ heterojunction was observed at various specific pump and probe wavelengths, demonstrating efficient charge separation to generate photocurrent. By increasing the annealing temperature of the Pc layer, we found that Pc thin films with higher crystallinity exhibited stronger SF and longer‐lived carrier transport, which was attributed to the increased stability and diffusion length. Finally, several SF solar cells were fabricated, in which the Pc layer exhibited high IQE, up to 160 ± 25% in the Pc layer, as modelled from the experimental EQE. Thus the SF process demonstrated significant contribution to the EQE of devices with thin films of AgBiS_2_ nanocrystals. Further engineering to increase the AgBiS_2_ thickness in n‐i‐p and p‐i‐n architectures, without sacrificing SF absorbance and carrier mobility, is needed to realize the full potential of SF in AgBiS_2_ solar cells and increase the PCE. However, we believe that these results suggest a reasonable pathway to eventually surpass the Shockley–Queisser limit and incorporate the SF mechanism into solar cell using nontoxic nanocrystalline materials.

4

TEM image of AgBiS_2_ NCs; UPS of AgBiS_2_ and pentacene; TA maps of samples prepared at room temperature with 750 nm pump laser. SEM and AFM images of pentacene film annealed at 100 °C; TA maps of sample annealed at 80 °C with 650 nm pump laser. The evolution of TA spectra for all sample with 650 nm pump laser; Device data of AgBiS_2_/Pc with different annealing temperature; IQE modeling details; Device data of p‐i‐n AgBiS_2_ SF solar cells; Fitting parameters for TA spectroscopy.

## Conflict of Interest

The authors declare no conflict of interest.

## Author Contributions

P.G. and D.C. contributed equally to this work. P.G. performed TA characterization and data analysis. D.C. performed the material synthesis, the device fabrication and the optical characterization. P.G. and D.C. wrote the manuscript and produced the figures. S.B.S. contributed to the key idea, performed TEM, SEM, and AFM characterization, and the early works of device fabrication. X.C. provided the equipment for TAS and related guidance. L.G. and J.E.H. supervised the project, analyzed the data and edited the manuscript. All authors contributed to the results and discussion, and edited the manuscript.

## Supporting information

Supporting Information

## Data Availability

The data that support the findings of this study are available in the supplementary material of this article.
