# Supplementary material for: Carrier Dynamics of Efficient Triplet Harvesting in AgBiS2/Pentacene Singlet Fission Solar Cells
Source: Adv Sci (Weinh). 2023 Mar 20;10(13):2300177. doi: 10.1002/advs.202300177 (PMC10161067; doi:10.1002/advs.202300177)
Supplement: Supplementary file 1 — Supporting Information [file ADVS-10-2300177-s001.pdf]

Supporting Information

Carrier Dynamics of Efficient Triplet Harvesting in $AgBiS_2$/Pentacene Singlet Fission Solar Cells

Pai Geng[#], Dezhang Chen[#], Sunil B. Shivarudraiah, Xihan Chen, Liang Guo, and Jonathan E. Halpert**

Experimental Section

Materials and Chemicals

Bismuth(III) acetate ($Bi(OAc)_3$, 99.999%), oleic acid (OA, 90%), hexamethyldisilathiane (HMS, synthesis grade), octadecene (ODE, 90%), tetramethylammonium iodide (TMAI, 99%), zinc acetate dihydrate ($Zn(OAc)_2{\cdot}2H_2O$, 98%), ethanolamine (MEA, 99.0%), Molybdenum(VI) oxide (MoO_3, 99.97%), toluene (anhydrous, 99.8%), pentacene (99.9%), 1,2-dichlorobenzene (DCB, anhydrous, 99%) were purchased from Sigma-Aldrich. 2-methoxyethanol (ACS reagent, 99.5%) was purchased from Merck. Methanol (MeOH, HPLC, 99.9%) were purchased from RCI Labscan. Silver acetate (AgOAc, 99%), poly[[4,8-bis[(2-ethylhexyl)oxy]benzo[1,2-*b*:4,5-*b*′]dithiophene-2,6-diyl][3-fluoro-2-[(2-ethylhexyl)carbonyl]thieno[3,4-*b*]thiophenediyl]] (PTB7) were purchased from J&K Chemical. All the chemicals were purchased and used without further purification.

Synthesis of $AgBiS_2$ Nanocrystals

$AgBiS_2$ NCs were synthesized using previously reported routes[1]. In brief, in a 3-neck round bottom flask, 1 mmol $Bi(OAc)_3$ and 0.8 mmol Ag(OAc) were dissolved in 6 mL OA and 4.5 mL ODE mixture. The mixture was raised to 100˚C under vacuum and degassed overnight. Then the atmosphere was switched to N_2 and 1 mmol HMS mixed with 0.5 mL degassed ODE was swiftly injected into the flask. The heating was stopped and the reaction cooled slowly without removing the heating mantel. The nanocrystals were isolated after adding 25 mL acetone and centrifugation, and redispersed in toluene. The purification process was performed twice, and the NCs were finally dispersed in anhydrous toluene and centrifuged. The NCs supernatant (20 mg / mL) was filtered by a 0.45 μm PTFE filter and stored in ambient atmosphere.

Thin-film Preparation for TA Spectroscopy

All the thin films were deposited on ITO substrates. For $AgBiS_2$ samples, a layer-by-layer (LBL) process was performed. Each LBL cycle consists: i) casting 1 drop of $AgBiS_2$ NCs on ITO substrates and spinning coating for 10 s at 2000 rpm. ii) casting 5 drops of TMAI solution (1 mg mL^{-1} in methanol) and waiting for 20 s, and spinning to dry. iii) repeating step ii once. iv) rinsing the film with methanol and toluene sequentially, and spinning to dry. After the deposition, the samples were annealed at 100 ˚C for 15 min. For pentacene samples, 25 nm of pentacene was thermally evaporated by a thermal evaporator. During the evaporation, the substrate temperature was set to 30 ˚C, 60 ˚C and 80 ˚C for samples with different annealing temperature. Samples were all stored in glovebox before optical characterizations.

Solar Cell Fabrication

The devices were fabricated by previously reported methods with modifications[2]. In brief, patterned ITO substrates were sequentially cleaned with Micro-90, deionized water, acetone and isopropanol under 20 minutes of sonication, and dried in an oven overnight. 10 minutes O_2 plasma was performed to remove the organic residue on ITO chips. 1.0 g zinc acetate dihydrate was dissolved in 10 mL 2-methoxyethanol and 284 μL ethanolamine. The solution is filtered by a 0.45 μm PTFE filter, and spin-casted to the ITO substrates at 3000 rpm for 30s and the substrates are annealed at 200 ˚C for 30 mins. The ZnO deposition process is repeated once to have ~35 nm thick compact ZnO films. Then the substrates were transferred into an N_2 purged dry box (RH ~20%), and the layer-by-layer (LBL) process was performed up to three times to obtain 1L / 2L $AgBiS_2$ samples, respectively. After the deposition, the samples were annealed at 100 ˚C for 15 min. For control devices, PTB7 solution (5 mg mL^{-1} in 1,2-dichlorobenzene) was spun on the samples at 2000 rpm for 30 s. For singlet fission devices, 25 nm of pentacene is thermally evaporated by a thermal evaporator. During the evaporation, the substrate temperature is set to 30 ˚C (RT), 60 ˚C and 80 ˚C for different annealing temperature. Lastly, 3 nm of MoO_3 and 100 nm of Ag was evaporated on the substrate with masks (active area = 7 mm^2). For p-i-n devices, the control devices were fabricated by the previously reported methods[3]. The p-i-n singlet fission (SF) devices use evaporated pentacene at 80 ˚C instead of spray-coated NiO, while the other procedures were identical.

Characterization

Ultraviolet-visible (UV-vis) absorption spectra were recorded using a Nanbei NU-T5 UV/Vis spectrophotometer. The crystalline structure of the $AgBiS_2$ and pentacene films was

determined via an x-ray diffractometer (XRD, PW1825 Philips). TEM images were obtained using JEOL 2010 microscope with an acceleration voltage of 200 kV. Samples were prepared by drop-casting dilute $AgBiS_2$ dispersions onto carbon-coated copper grids. The morphologies of the $AgBiS_2$ and pentacene films were characterized by a field- emission scanning electron microscope (SEM; JEOL 7100F) operated at 5 kV. Atomic force microscopy (AFM) was measured using a Park System NX10 microscope in tapping mode. Ultraviolet photoelectron spectroscopy (UPS) was measured using a Kratos Axis Ultra with base pressure ~ 4×10^{-10} mbar equipped with a He-I UV-light source (21.22 eV). The current density-voltage (J-V) curves of the devices were obtained with a Keithley 2400 digital source meter under simulated AM 1.5G spectrum at 100 mW cm^{-2}, with a solar simulator (Class AAA, 94023A-U; Newport Corporation). The active area of the device was defined as 0.056 cm^{-2} through a shadow mask. External quantum efficiency spectra were measured using an EQE (Enlitech QS) system equipped with a standard Si diode with the monochromatic light generated from a Newport 300 W lamp source.

Transient Absorption (TA) Spectroscopy

The transient absorption measurements are performed by a pump-probe spectrometer (TA-100, Time-tech spectra). Fundamental laser with a center wavelength of 800 nm and pulse duration around 150 fs is generated at 1 kHz repetition rate by a Ti:Sapphire amplifier (Astrella, Coherent). Then the fundamental laser is split into two parts, one part is guided into an optical parameter amplifiers (OPA, TOPAS, Light Conversion) to create a pump laser with variable wavelength, whose frequency is chopped at 500 Hz. The other part of laser is focused into a sapphire crystal or another thick sapphire crystal, to generate 450-810 nm or 750-1500 nm probe laser, respectively. A motorized translation stage is used in the optical path of the probe laser to achieve the time delay with femtosecond resolution. In this experiment of 650 nm and 750 nm pump wavelentghs are adopted, with pulse energy 200 nJ and 160 nJ, respectively. When testing the composite film, the $AgBiS_2$ layer is placed on the side facing the laser, to match the situation of the acutal application.

IQE Calculation:

The optical constants (refractive indices, n and extinction coefficients, k) were measured by a spectroscopic ellipsometer (Alpha-SE, J.A. Woollam). The measured sample were prepared by spin-coating or evaporating the materials on silicon substrates. For ITO, glass, MoO_x, and Ag, the optical constants were obtained from the public database. For ZnO, PTB7 and

pentacene, the film thicknesses and optical constant were fitted with a Cauchy model on their transparent regions. For $AgBiS_2$, the absorbing layer on Silicon model was used and B-spline fitting was used. The 1L, 2L, 3L samples are measured and compared with absorbance spectra to validate the fitting. We used the following parameters in the transfer matrix optical modeling[4], which the actual layer thickness has been validated by surface profiler (Alpha-Step D300, KLA Tencor) and ellipsometer:

**Thickness (nm) used in transfer matrix optical modelling**						
**Sample**	**ITO**	**ZnO**	**$AgBiS_2$**	**PTB7 / Pc**	**MoO_3**	**Ag**
PTB7-1L	200	40	17	10	3	100
Pc-1L	200	40	17	25	3	100
PTB7-2L	200	40	25	10	3	100
Pc-2L	200	40	25	25	3	100

The absorbing fractions generated by the model were used to calculated the IQEs.

The overall IQE in the SF device:

$$IQE_{AgBiS_2/Pc} = \frac{EQE_{AgBiS_2/Pc}}{Abs_{AgBiS_2} + Abs_{Pc}}$$

The pentacene EQE components in the device were calculated by subtracting the modelled $AgBiS_2$ EQE fraction by the overall EQE. The pentacene IQE in the SF device:

$$IQE_{Pc} = \frac{EQE_{AgBiS_2/Pc} - EQE_{AgBiS_2\,(Modeled)}}{Abs_{Pc}}$$

It is note worthy that for a very small absorption fraction of pentacene (400 nm to 500 nm range, where pentacene barely absorbs), the IQE calculated will have large error. Therefore, only the range that pentacene strongly absorbs (500 nm to 750 nm) is considered in the IQE calculation.

For top-absorbing solar cell modelling, all the layer positions were reversed except for ITO/Ag layer.

All the modelled absorbance fractions and calculated EQEs, IQEs are plotted in Figure S8-10

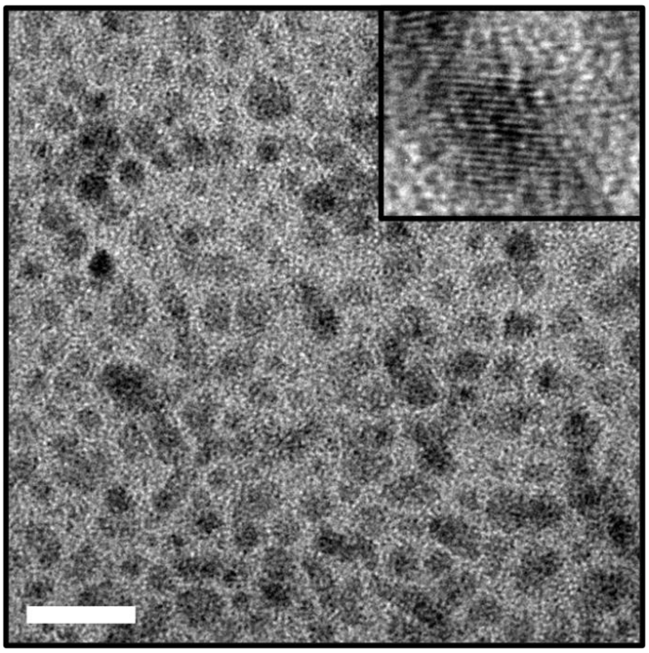

Figure S1. TEM image of $AgBiS_2$ nanocrystals. Inset shows the crystal lattice of $AgBiS_2$ NCs. Scale bar is 20 nm.

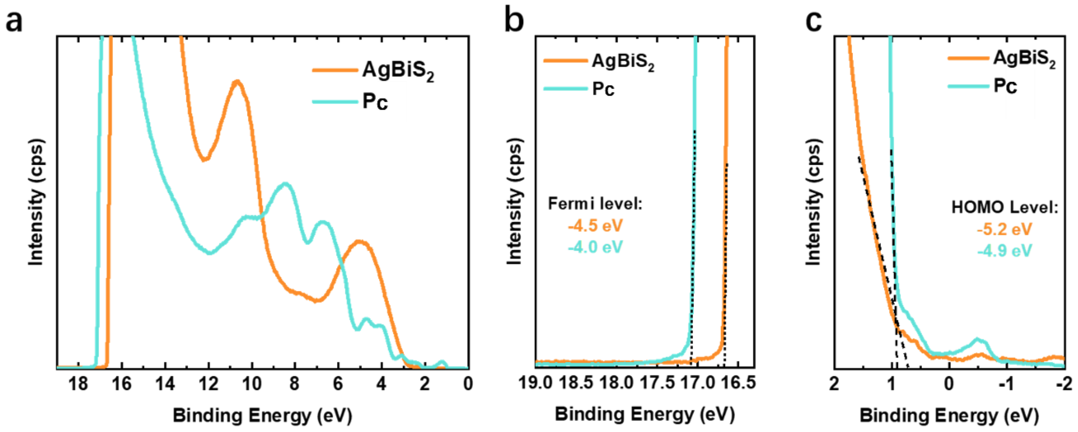

Figure S2. (a) UPS of $AgBiS_2$ and pentacene. (b) Fermi level of $AgBiS_2$ and pentacene. (c) HOMO level of $AgBiS_2$ and pentacene.

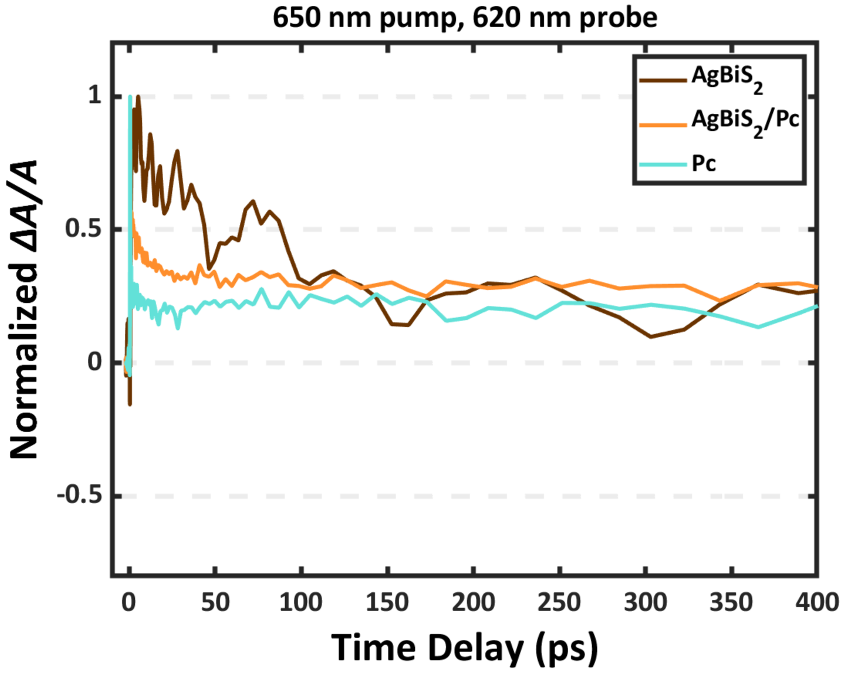

Figure S3. TA spectra for all samples with 650 nm pump laser and 620 nm probe laser.

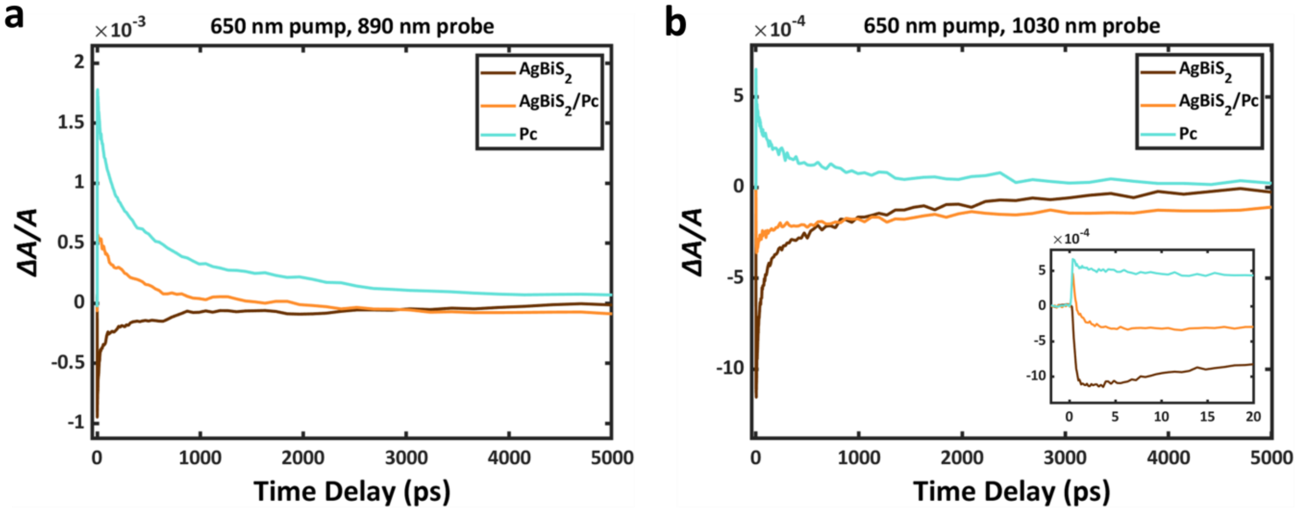

Figure S4. (a) TA spectra for all samples with 650 nm pump laser and 890 nm probe laser. (b) TA spectra for all samples with 650 nm pump laser and 1030 nm probe laser. Insets shows the TA evolution in 20 ps.

Under 650 nm pump and 890 nm probe, the triplets in Pc exhibits strong PIA signals, since there are no triplet acceptors and the recombination of triplets and holes is spin-forbidden, the triplet PIA signals will remain for a long time. However, in the composite sample, this PIA signal will revert to 0 in a short time, since the triplet transferred to $AgBiS_2$ will occupy its conduction band, the GSB of $AgBiS_2$ will be enhanced and finally the TA signal for the composite sample at this wavelength exhibit a negative $\Delta A/A$.

Similarly, while probing at 1030 nm, the $AgBiS_2$ will exhibit a strong GSB signal. For the pure $AgBiS_2$ sample, this GSB signal will revert to 0 in 5 ns. For the composite sample, the long-lived triplets will transfer into the $AgBiS_2$, result in an enhanced GSB signal that can stay for long time (> 5 ns). The inset display the evolution of 1030 nm TA signal in first 20 ps, and the triplet transfer can be easy to track. The Dexter triplet transfer from Pc to $AgBiS_2$ occurs in ~2 ps, which is much faster than the hole transfer from $AgBiS_2$ to Pc (we can observe this process through Figure 3c), so the triplet transfer induced bleach recovery should be convincing.

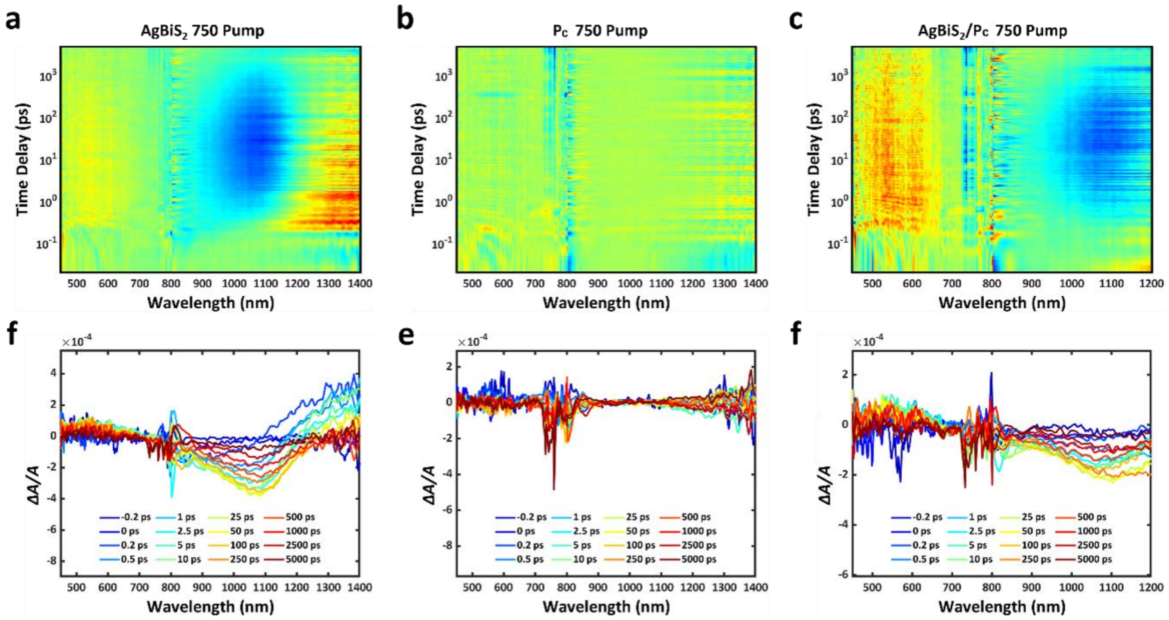

Figure S5. TA maps of (a) $AgBiS_2$, (b) Pc and (c) $AgBiS_2$/Pc. The evolution of TA spectra with different time delay for (d) $AgBiS_2$, (e) Pc and (f) $AgBiS_2$/Pc, respectively. The pump wavelength is 750 nm.

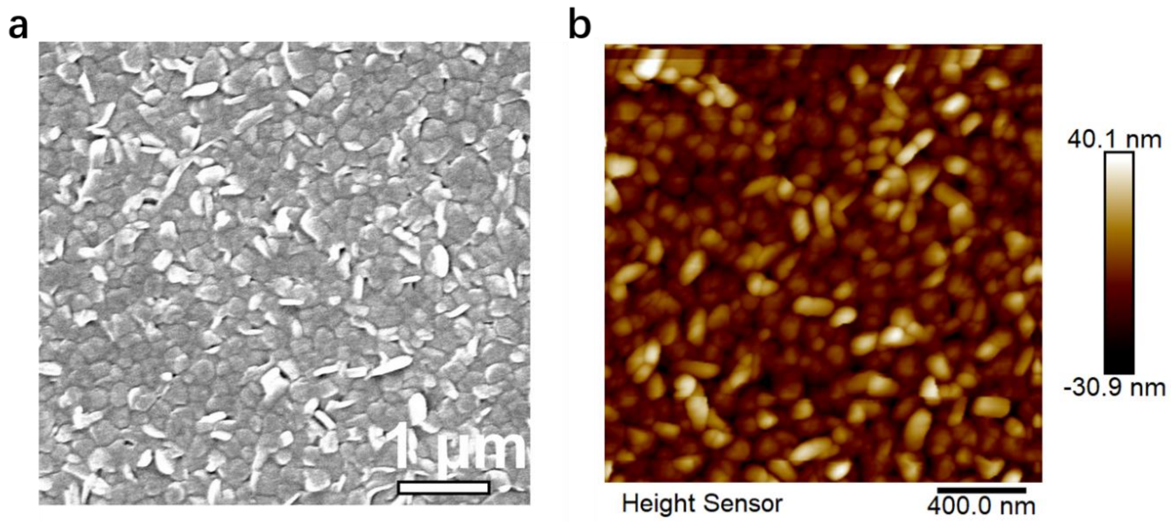

Figure S6. (a) SEM and (b) AFM image of pentacene film annealed at 100 °C. Pinholes and roughness are increased greatly, which negatively affects the photovoltaic performances.

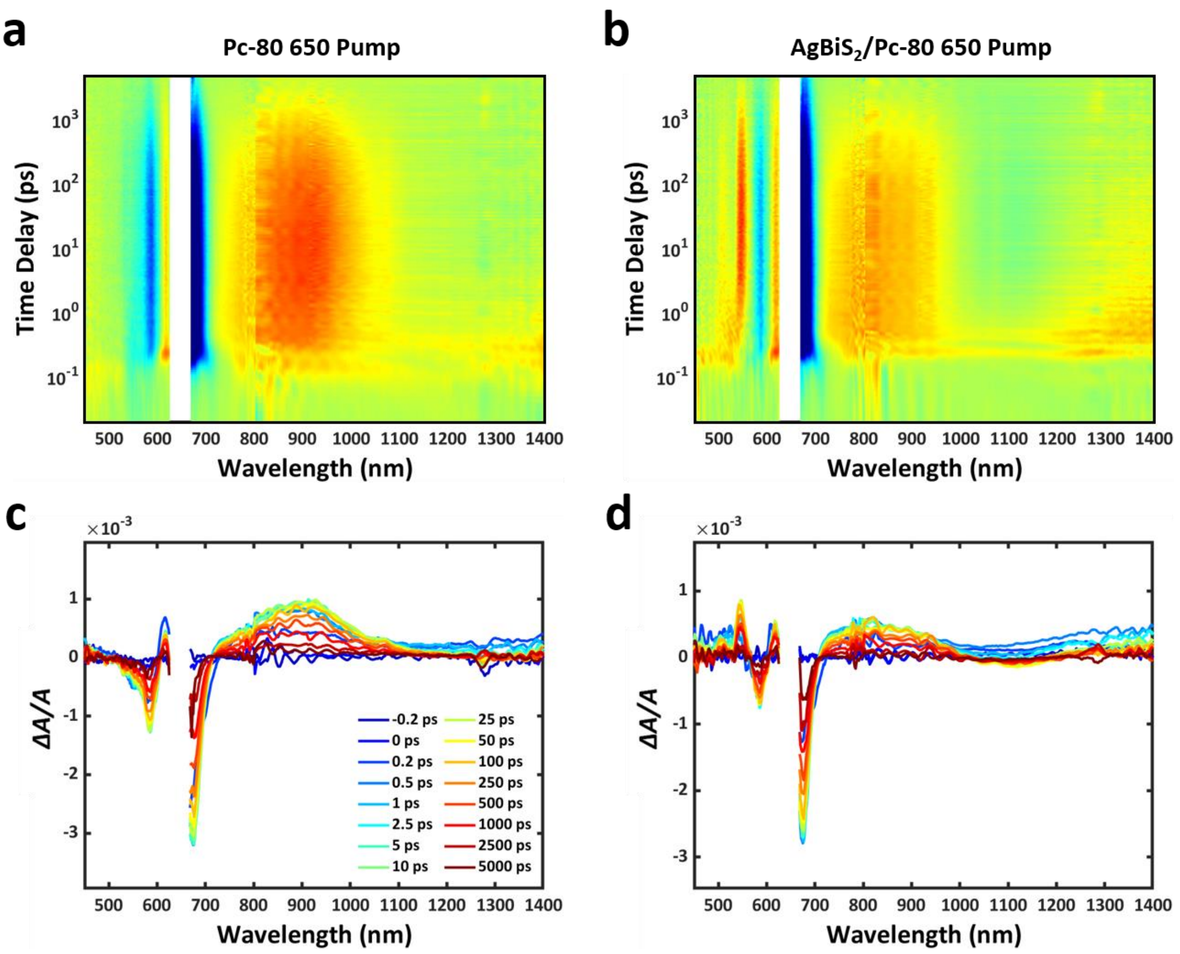

Figure S7. TA maps of (a) Pc-80 and (b) $AgBiS_2$/Pc-80. The evolution of TA spectra with different time delay for (c) Pc-80 and (d) $AgBiS_2$/Pc-80, respectively. The pump wavelength is 650 nm.

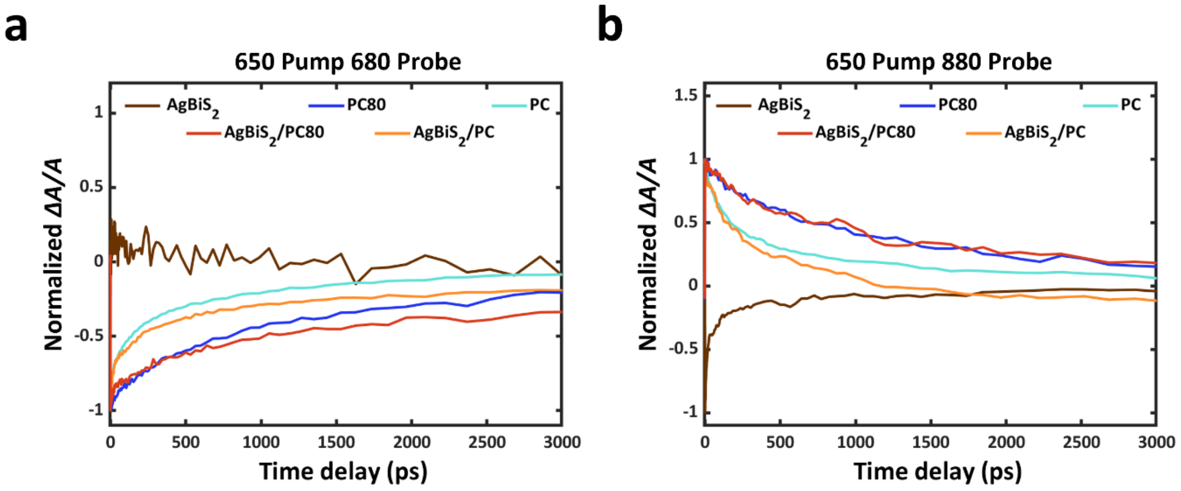

Figure S8. The evolution of TA spectra with different time delay for all sample with pump wavelength as 650 nm and probe wavelength as (a) 680 nm and (b) 880 nm, respectively. Both the GSB and the PIA signal of Pc-80 or $AgBiS_2$/Pc-80 sample exhibit longer lifetime than the normal Pc or $AgBiS_2$/Pc sample.

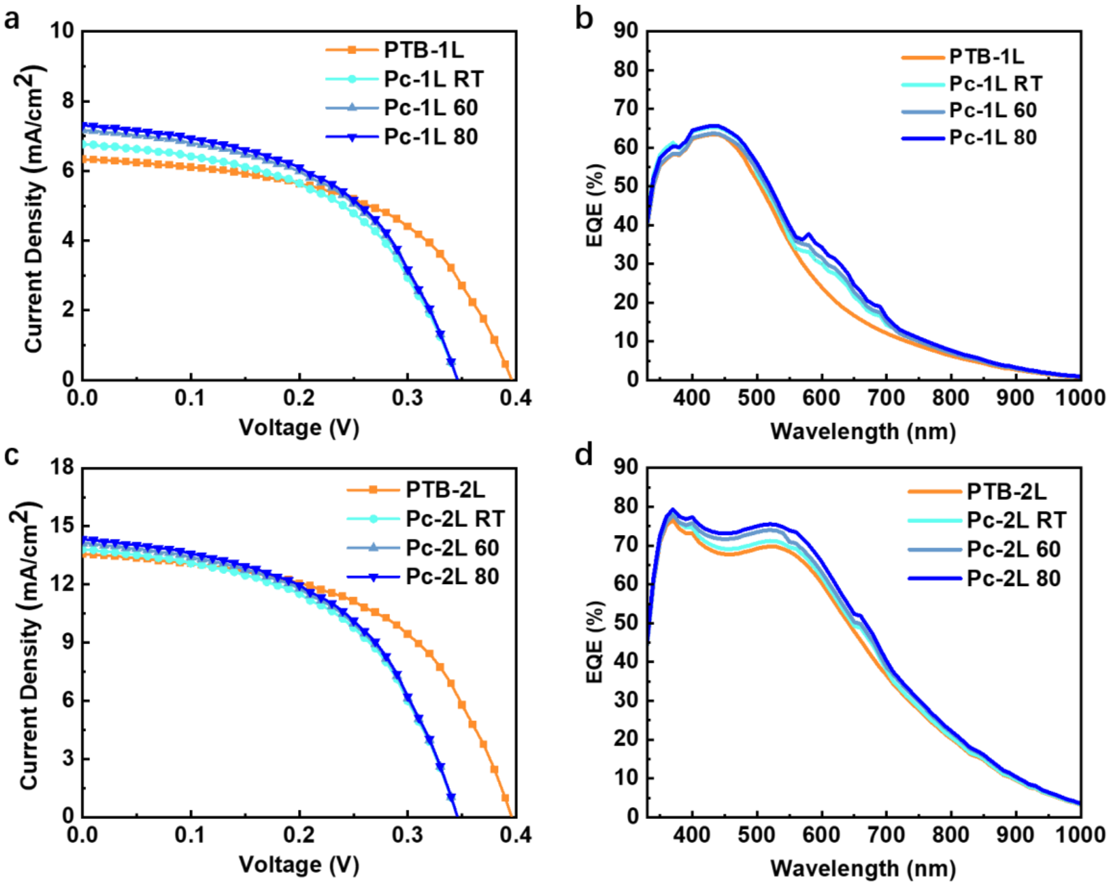

Figure S9. *J-V* curve and EQE curves of $AgBiS_2$/Pc with different Pc annealing temperature.

**Summary of SF $AgBiS_2$ solar cell's photovoltaic performance**				
**Devices**	**V_{OC} (V)**	**J_{sc} (mA cm^{-2})**	**FF**	**PCE (%)**
PTB-1L	0.395	6.345	0.541	1.355
Pc-1L RT	0.335	6.854	0.523	1.200
Pc-1L 60	0.332	7.077	0.526	1.235
Pc-1L 80	0.322	7.374	0.530	1.250
PTB-2L	0.395	13.501	0.541	2.885
Pc-2L RT	0.335	13.895	0.523	2.434
Pc-2L 60	0.332	14.142	0.526	2.469
Pc-2L 80	0.322	14.342	0.530	2.447

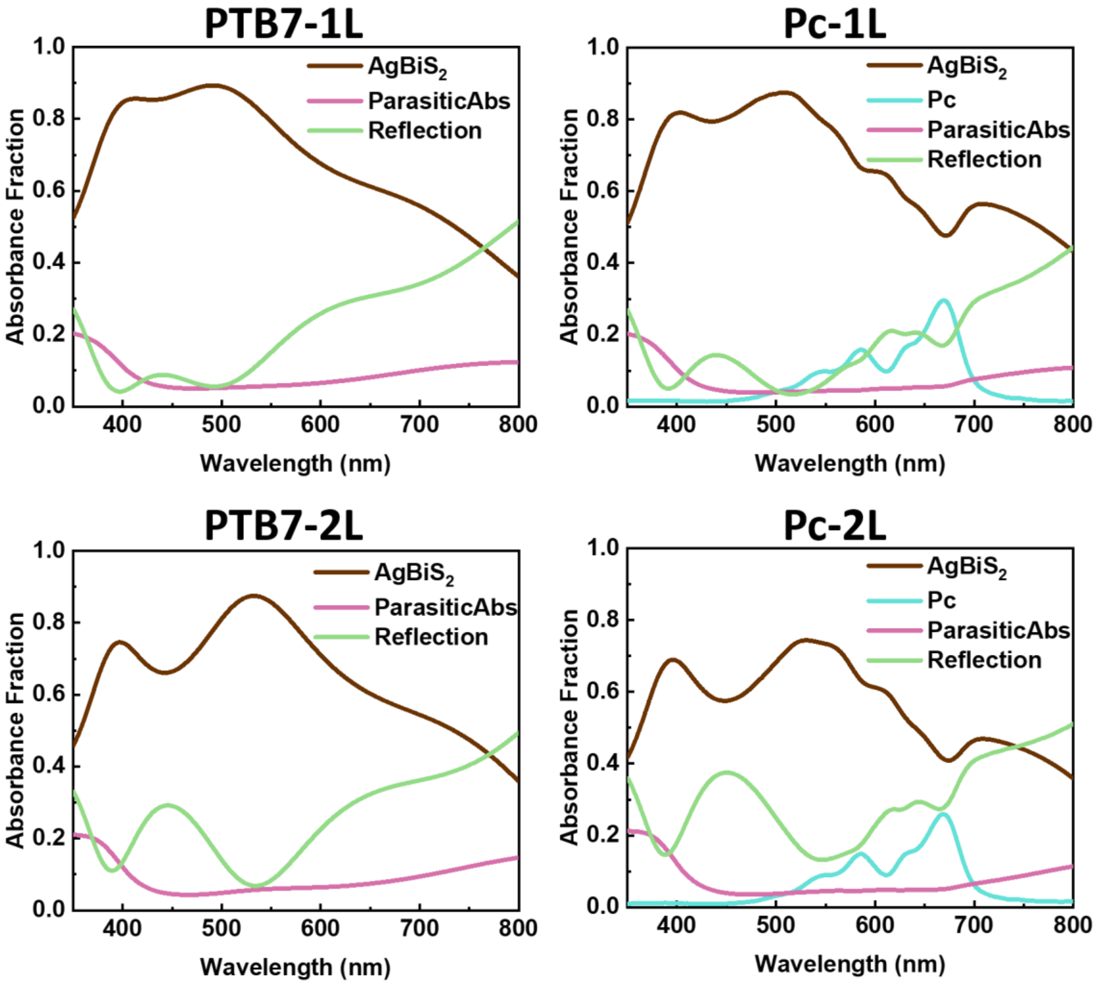

Figure S10. Absorbance fraction in the control and SF devices, respectively. The parasitic absorption (absorbance from non-active layer) (pink) and reflection (green) inside the devices are considered.

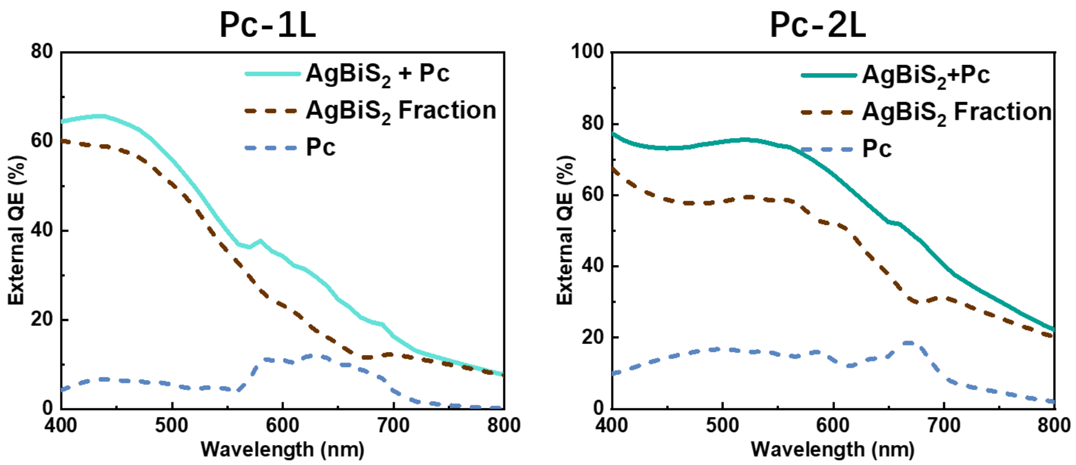

Figure S11. Modelled EQE fraction from 1L / 2L SF devices.

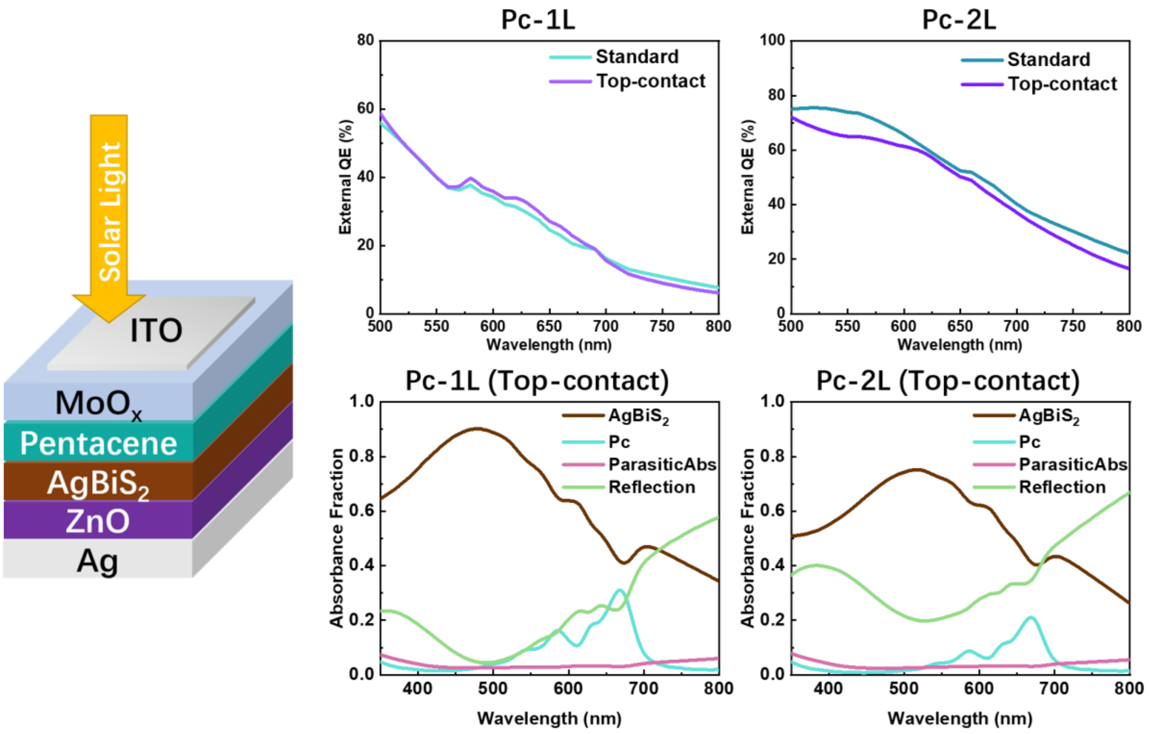

Figure S12. Top-contact SF device structure, modelled EQEs, and modelled light fraction inside the devices. Overall higher reflection fraction (green) is observed in top-contact Pc-2L sample compared with the control samples in Figure S8, resulting in lower modelled EQE in Pc-2L samples.

Improving the singlet fission and PCE via device engineering

To fabricate a practically efficient SF solar cell, the incident light needs to pass the SF material before reaching the active layer. However, in a thick n-i-p structure, over 70% of the light is absorbed before reaching the Pc film (**Figure S10**). Combined with the low carrier mobility inside the thick $AgBiS_2$ film, thicker $AgBiS_2$ solar cells did not effectively demonstrate strong contribution from SF. Based on the EQE achieved in the n-i-p devices, we simulated the EQE of a top-contact SF $AgBiS_2$ device (**Figure S12**). The simulated 1L device shows higher modelled EQE in the Pc absorbing range, suggesting a theoretical advantage in the top-contact structure. However, at longer wavelengths (>700 nm), the top-contact SF device shows lower EQE than the normal device; this is attributed to the lower absorption of Pc at this range. The 2L top contact device shows a higher reflectance of the incident light, which further decreases the overall EQE (**Figure S12**). We conclude that although the optimized structure may utilize the incident light more efficiently, the reflection in the heterojunction cannot be ignored, and a textured surface that minimizes the reflection effect needs to be developed. To further investigate the SF effect in these materials, we also fabricated p-i-n type SF devices based on our previous research, where Pc is evaporated before the deposition of the $AgBiS_2$ layers. The device architecture for the p-i-n device was ITO/Pc/$AgBiS_2$/PCBM/BCP/Ag (**Figure S13**). However, an unexpected low device performance was observed in all the p-i-n SF devices which is attributed to Pc film damage during the critical NC ligand-exchange process using polar solvents. We suggest that the future development of $AgBiS_2$ one-step deposition or a top-contact structure will be needed for practical p-i-n solar cells.

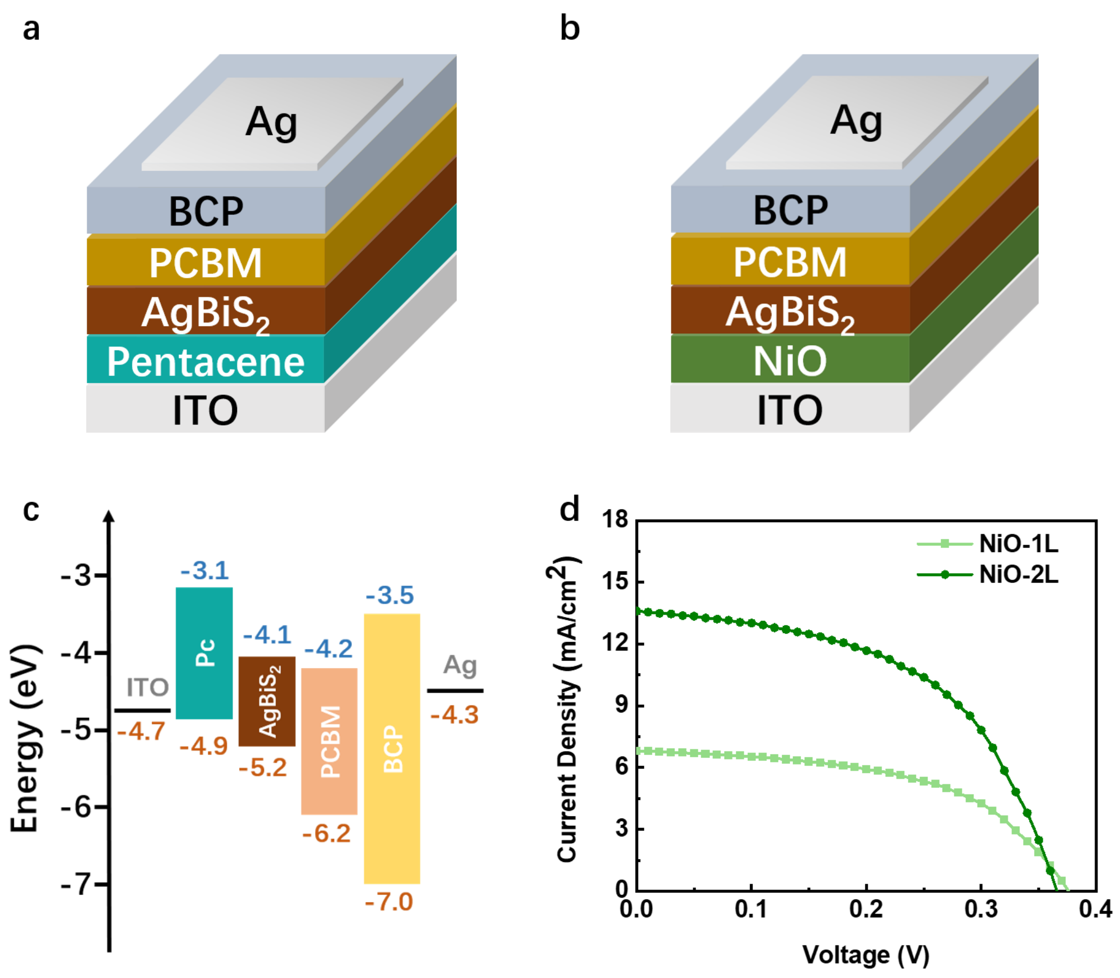

Figure S13. Device structure of (a) designed p-i-n SF $AgBiS_2$ device and (b) p-i-n control device. (c) p-i-n SF device band energy diagram. (d) *J-V* curves of the control devices, SF device didn't work and thus not shown in the figure.

**Summary of p-i-n $AgBiS_2$ solar cell's photovoltaic performance.**				
**Devices**	**V_{OC} (V)**	**J_{sc} (mA cm^{-2})**	**FF**	**PCE (%)**
NiO-1L	0.370	6.801	0.593	1.492
NiO-2L	0.350	13.680	0.593	2.823
p-i-n Pc-1L	0.00082	1.289	0.000	0.000
p-i-n Pc-2L	0.01562	3.599	0.229	0.013
p-i-n Pc-3L	0.05610	3.581	0.225	0.045

**Sample / Signal**	$A_{singlet}$ **or** $A_{triplet}$	t_0 **(ps)**	y_0	$\tau_{singlet}$ **or** $\tau_{triplet}$ **(ps)**
Pc-RT / Singlet PIA	1.034	0.1702	0.09347	0.1184±0.022
Pc-RT / Triplet PIA	0.9084	0.1072	-0.01376	0.1284±0.010
Pc-80 / Singlet PIA	0.996	0.1739	0.0511	0.08813±0.023
Pc-80 / Triplet PIA	1.002	0.113	-0.1105	0.1189±0.036

Table S1. Time constant and fitting parameters for TA spectroscopy.

[1] Y. Wang, S. R. Kavanagh, I. Burgués-Ceballos, A. Walsh, D. O. Scanlon, G. Konstantatos, *Nat. Photonics* **2022**, *16*. 235.
[2] M. Bernechea, N. C. Miller, G. Xercavins, D. So, A. Stavrinadis, G. Konstantatos, *Nat. Photonics* **2016**, *10*. 521.
[3] D. Chen, S. B. Shivarudraiah, P. Geng, M. Ng, C. H. A. Li, N. Tewari, X. Zou, K. S. Wong, L. Guo, J. E. Halpert, *ACS Appl. Mater. Interfaces* **2022**, *14*. 1634.
[4] G. F. Burkhard, E. T. Hoke, M. D. McGehee, *Adv. Mater.* **2010**, *22*. 3293.
